# Inhibitory effect of *Streptococcus salivarius* K12 and M18 on halitosis in vitro

**DOI:** 10.1002/cre2.269

**Published:** 2019-12-23

**Authors:** Hyun‐Jun Yoo, Su‐Kyung Jwa, Da‐Hui Kim, Yun‐Jeong Ji

**Affiliations:** ^1^ Department of Preventive Dentistry Dankook University College of Dentistry Republic of Korea; ^2^ Department of Dental Hygiene Ulsan College Republic of Korea; ^3^ Herbal Crop Utilization Research Team, Department of Herbal Crop Research Rural Development Administration (RDA) Chungbuk Republic of Korea

**Keywords:** oral malodor, *Streptococcus salivarius* K12, *Streptococcus salivarius* M18

## Abstract

**Background:**

The aim of the study was to observe the antimicrobial activity of *Porphyromonas gingivalis* and *Treponema denticola* as well as the effect on reducing volatile sulfur compounds (VSCs).

**Materials and methods:**

After *P. gingivalis* and *T. denticola* were cultured with or without *Streptococcus salivarius* K12 and M18, VSCs were measured by Oral Chroma. In order to analyze the mechanism for malodor control, the antimicrobial activity of *S. salivarius* K12 and M18 against *P. gingivalis* and *T. denticola* was assessed. SPSS 21.0 was used for data analysis with the Kruskal–Wallis and Jonckheere–Terpstra tests. Mann–Whitney test was applied for post hoc analysis.

**Results:**

*P. gingivalis* and *T. denticola* VSC levels were reduced by high concentrations of *S. salivarius* K12 and M18 during coculture. The concentrations were lower than those of single culture (*p* < .05). An antimicrobial effect was detected on *P. gingivalis*, and *T. denticola* by 50% *S. salivarius* K12 and M18. The spent culture medium and whole bacteria of *S. salivarius* K12 and M18 reduced the levels of VSCs below the amount in a single culture of *P. gingivalis* and *T. denticola* (*p* < .05).

**Conclusion:**

*S. salivarius* K12 and M18 decreased the levels of VSCs originating from *P. gingivalis* and *T. denticola.*

## 1 INTRODUCTION

Oral malodor is an unpleasant smell that occurs in the oral cavity and the nearby organs (Rosenberg et al., 1991). It is a foul odor that causes discomfort in others during expiration. In other words, oral malodor is defined not only as a foul odor that arises within the oral cavity, but also includes all unpleasant smells that pass through the oral cavity from other organs such as the stomach, the liver, and the lungs (Paik, Shin, Cho, Jang, & Lee, 2011).

Oral malodor has many potential causes, but 80–90% of cases occur because of factors within the oral cavity. It is usually the work of Gram negative anaerobic bacteria, especially *Porphyromonas gingivalis*, *Treponema denticola*, *Tannerella forsythia*, *Fusobacterium* spp. (*Fusobacterium nucleatum*, *Fusobacterium fusiform*, *Fusobacterium polymorphum*), and *Prevotella intermedia* (Kim, 2008; Kishi et al., 2013). Of these, *P. gingivalis* and *T. denticola* are not only known to be related to periodontal diseases, but are also found on the tongue and are known to synthesize volatile sulfur compounds (VSCs) when attached to the dorsal side of the tongue (Kishi et al., 2013).

Probiotics are defined as “living bacteria that are beneficial to health when an appropriate amount is consumed” (Kopp‐Hoolihan, 2001). Nonpathogenic microorganisms such as yeast and lactobacilli exist in food and offer benefits to humans (Brown & Valiere, 2004). Probiotics can improve diseases such as diarrhea, enteritis, ulcerative colitis, deterioration of immunity, and hyperlipidemia. Recently, in dental medicine, there have been many studies on the effectiveness of using oral cavity probiotics against oral diseases including dental caries and halitosis (Burton et al., 2013). Comelli, Guggenheim, Stingele, and Neeser (2002) showed that *Streptococcus thermophilus* and *Lactococcus lactis* have preventive effects against dental caries due to their ability to weaken dental plaque. Burton, Chilcott, and Tagg (2005) reported that *Streptococcus salivarius* K12 reduces the synthesis of VSCs.

The purpose of this study is to research and analyze how probiotic bacteria such as *S. salivarius* K12 and M18 affect the synthesis of sulfur compounds and the growth of *P. gingivalis* and *T. denticola*, which cause oral malodor.

## 2 MATERIALS AND METHODS

### 2.1 Bacteria and bacterial culture

The major bacteria used in this experiment are the bacteria associated with oral malodor, *P. gingivalis* ATCC 33277 and *T. denticola* ATCC 35405, which were purchased from American Type Culture Collection (ATCC). *P. gingivalis* was cultured in brain heart infusion (BHI) liquid media, which included hemin (0.05 μg/mL; Sigma, St Louis, MO) and Vitamin K (1 μg/mL; Sigma). *T. denticola* was cultured in tryptone‐yeast extract‐gelatin‐volatile fatty acids‐serum (TYGVS) media in a 37°C anaerobic state (5% H_2_, 10% CO_2_, 85% N_2_) (Ohta, Makinen, & Loesche, 1986). *S. salivarius* K12 and M18 (Thera Breth; The California Clinics, Los Angeles, CA) were used as probiotics and cultured in BHI media at 37°C. For the coculture of oral cavity bacteria, *P. gingivalis* was cultured in BHI media containing hemin and vitamin K. *T. denticola* was cultured in a mixed media consisting of TYGVS liquid media and BHI liquid media in a one‐to‐one ratio at 37°C in an anaerobic condition.

### 2.2 Measurement of VSC synthesis in single culture or coculture of oral malodor causing bacteria with *S. salivarius*

After *P. gingivalis*, *T. denticola* alone or mixed incubation with *S. salivarius*, 1 ml of bacterial culture was transferred to a clean 50 ml conical tube. Afterwards, to explore the inhibitory mechanisms involved in the synthesis of VSCs, probiotics bacteria media was centrifuged at 7,000*g* for 10 min. The supernatant was sterilized by passing it through a polyvinylidene filter (Millipore Co., Belleica, MA) with the pore size of 0.22 μm. The precipitated *S. salivarius* was washed with a phosphate buffer. *P. gingivalis* or *T. denticola* media was mixed with 1 × 10^7^, 2 × 10^7^, or 3 × 10^7^ of the filter sterilized *S. salivarius* and transferred to clean conical tubes. After vortexing for 30 min with a Vortex mixer (GENIE II; Scientific Industries, Bohemia, NY), 1 mL of the air above the media was sucked into a 10‐mL syringe. The syringe was pulled to the 10 mL line to dilute the air 10 times. Using Oral Chroma (ABILIT Corp., Tokyo, Japan), the amount of H_2_S, CH_3_SH, and (CH_3_)_2_S was measured.

### 2.3 The antimicrobial level of *S. salivarius* K12 and M18 against bacteria causing oral malodor

*S. salivarius* K12 and M18 were cultured and centrifuged at 7,000*g* for 10 min. The antimicrobial level was tested using the supernatant. The antimicrobial susceptibility test followed the protocols from the Clinical and Laboratory Standards Institute (CLSI) (Bosy, 1997). In each well of a 96‐well polystyrene culture plate, 180 μL of BHI containing hemin and Vitamin K was added. Media of the two probiotics bacteria (180 μL) was added to the first well and serially diluted, each time by half, using a multipipette. After incubating for 36 hr under anaerobic conditions at 37°C, absorbance at 650 nm was measured with a spectrophotometer. To evaluate the effect of *S. salivarius* colonies inside the oral cavity, the bacteria were cocultured using millicel inserts (Millipore Co., Belleica, MA).

The bacteria causing malodor were inoculated on the inside of the millicell insert, and *S. salivarius* was inoculated on the outside. They were then incubated for 36 hr under anaerobic conditions at 37°C. Contamination of the bacteria was verified using a phase‐contrast microscope (Nikon, Tokyo, Japan). The images of the bacteria were also obtained using a phase‐contrast microscope (Nikon).

### 2.4 Testing for statistical significance

Statistical significance between the case and control groups was tested with SPSS 21.0 (SPSS Inc., Chicago, IL), using Kruskal–Wallis and Jonckheere–Terpstra tests. Mann–Whitney test was used for post hoc analysis. We used the *p* value of .05 to test for significance.

## 3 RESULTS

### 3.1 The inhibitory effects of coculture of oral malodor causing bacteria and *S. salivarius* on the synthesis of VSCs

#### 3.1.1 The inhibitory effects of coculture of *P. gingivalis* and *S. salivarius* on the synthesis of VSCs

**Figure 1:**
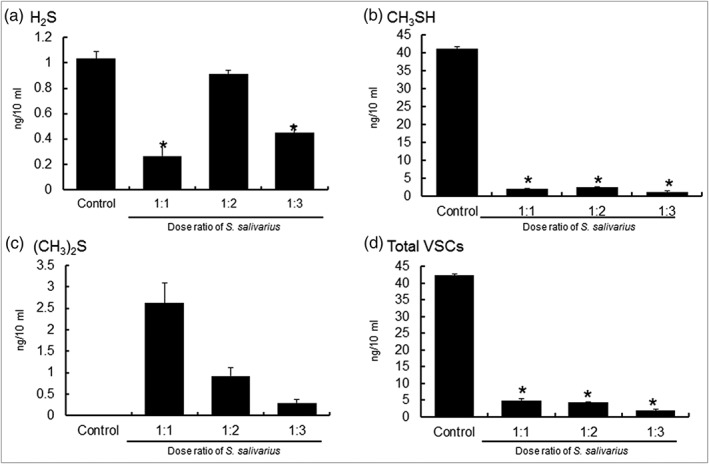
VSC of single‐cultured or cocultured *Porphyromonas gingivalis* with *Streptococcus salivarius* K12 and M18. VSC, volatile sulfur compound

After culturing *S. salivarius* by themselves or coculturing with oral malodor causing bacteria, Oral Chroma was used to measure the concentration of VSCs. When *P. gingivalis* and *S. salivarius* were cocultured, the level of VSCs that formed decreased significantly as the level of *S. salivarius* increased (Figure 1).

Table 1 shows the concentration of VSCs from a single culture of *P. gingivalis* or a coculture of *P. gingivalis* and *S. salivarius*.

**Table 1:** VSCs of single‐cultured or cocultured *Porphyromonas gingivalis* with *Streptococcus salivarius* K12 and M18

Group	HS (*n* = 7)		MM (*n* = 7)		DMS (*n* = 7)		VSCs (*n* = 7)	
	*M*	SD	*M*	SD	*M*	SD	*M*	SD
Control	1.03	0.31	41.19**	3.18	0.01	0.02	42.3**	3.46
1:1	0.27*	0.34	2.08*	1.41	2.63	3.28	4.97*	2.98
1:2	0.92	0.74	2.48*	1.13	0.92	1.34	4.32*	1.20
1:3	0.45*	0.38	1.27*	1.43	0.30	0.54	2.02*	1.42

*Note:* Unit: ng/10 mL.

Abbreviations: DMS, dimethyl sulfide; HS, hydrogen sulfide; MM, methyl mercaptan; VSC, volatile sulfur compound.

*p < .05 by Kruskal–Wallis test; **p < .05 by Jonckheere–Terpstra test.

The total concentration of VSCs was highest in the single culture of *P. gingivalis* with 42.32 ng/10 mL. When 1 × 10^7^ cells of *S. salivarius* were added, the total concentration dropped to 4.97 ng/10 mL. The concentration dropped to 4.32 and 2.02 ng/10 mL when 2 × 10^7^ and 3 × 10^7^ cells of *S. salivarius* were added, respectively. As the concentration of *S. salivarius* increased, the level of induction of VSCs for *P. gingivalis* decreased (*p* < .05).

#### 3.1.2 The inhibitory effects of coculture of *T. denticola* and *S. salivarius* on the synthesis of VSCs

**Figure 2:**
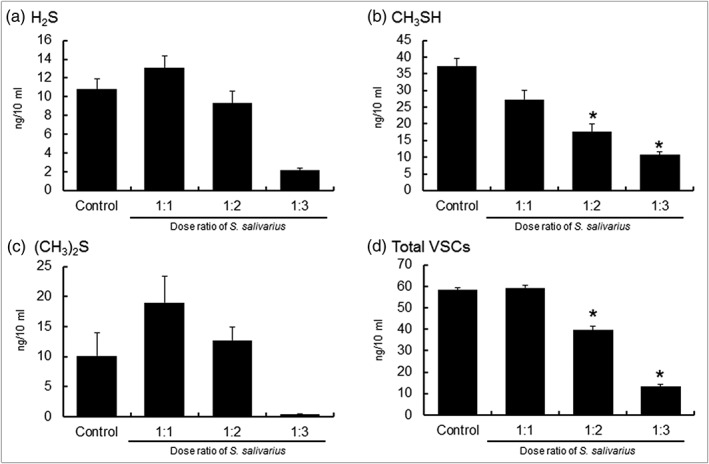
VSC of single‐cultured or cocultured *Treponema denticola* with *Streptococcus salivarius* K12 and M18. VSC, volatile sulfur compound

When *T. denticola* and *S. salivarius* were cocultured, the level of VSCs that formed decreased significantly as the level of *S. salivarius* increased (Figure 2).

Table 2 shows the concentration of VSCs from a single culture of *T. denticola* or a coculture of *T. denticola* and *S. salivarius*.

**Table 2:** VSC of single‐cultured or co‐cultured *Treponema denticola* with *Streptococcus salivarius* K12 and M18

Group	HS (*n* = 7)		MM (*n* = 7)	DMS (*n* = 7)			VSCs (*n* = 7)	
	*M*	SD	*M*	SD	*M*	SD	*M*	SD
Control	10.82	7.81	37.32**	16.46	10.17	26.90	58.30**	8.39
1:1	13.08	8.93	27.27	19.16	19.01	31.01	59.36	7.06
1:2	9.31	8.78	17.63*	16.69	12.69	16.23	39.64*	11.74
1:3	2.16	1.75	10.67*	6.38	0.43	0.34	13.25*	8.06

*Note:* Unit: ng/10 mL.

Abbreviations: DMS, dimethyl sulfide; HS, hydrogen sulfide; MM, methyl mercaptan; VSC, volatile sulfur compound.

*p < .05 by Kruskal–Wallis test; **p < .05 by Jonckheere–Terpstra test.

The concentration of methyl mercaptan was highest at 37.32 ng/10 mL when *T. denticola* were cultured by themselves. When 1 × 10^7^, 2 × 10^7^, and 3 × 10^7^ cells of *S. salivarius* were added and cocultured, the concentration dropped to 27.27, 17.63, and 10.67 ng/10 mL, respectively (*p* < .05). The total concentration of VSCs, when *T. denticola* were cultured by themselves, 58.30 ng/10 mL, was similar to the total concentration when *T. denticola* and 1 × 10^7^ cells of *S. salivarius* were cocultured, 59.37 ng/10 mL. However, with 2 × 10^7^ and 3 × 10^7^ cells of *S. salivarius*, the total concentration dropped to 39.64 and 13.25 ng/10 mL, respectively (*p* < .05).

### 3.2 The antimicrobial activity of *S. salivarius* K12 and M18 on oral malodor causing bacteria

**Figure 3:**
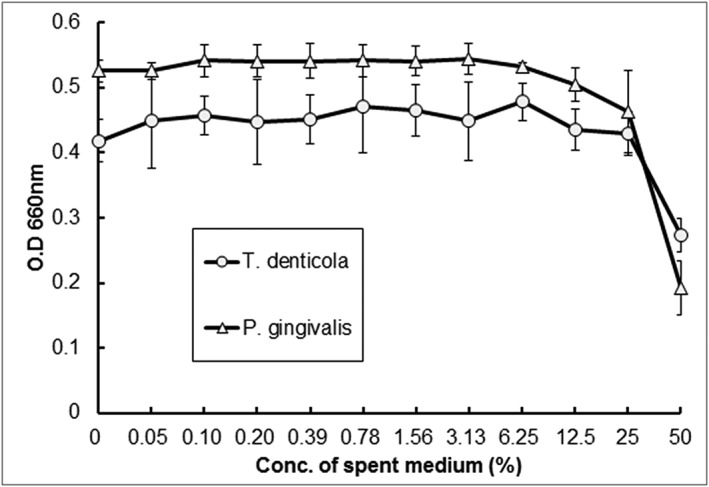
Antimicrobial activity of *Streptococcus salivarius* K12 and M18 against oral malodor generating bacteria

The synthesis of VSCs by oral malodor causing bacteria decreased in the presence of *S. salivarius* K12 and M18. To investigate the mechanism underlying *S. salivarius'* inhibition of the synthesis of VSCs by oral malodor causing bacteria, an antimicrobial activity test was performed. When the antimicrobial activity test recommended by CLSI was performed, *S. salivarius* K12 and M18 were found to have antimicrobial activity against *P. gingivalis* and *T. denticola* (Figure 3).

**Figure 4:**
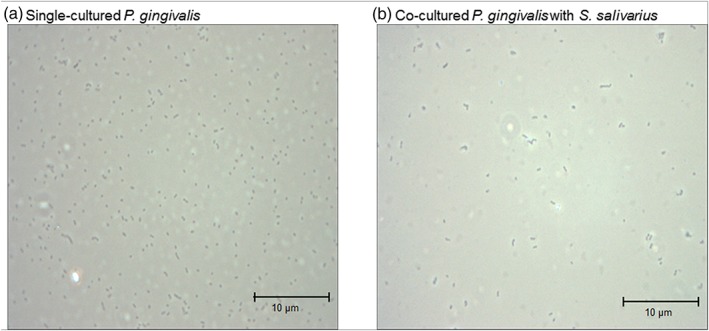
Phase‐contrast microscopic image of single‐cultured or cocultured *Porphyromonas gingivalis* with *Streptococcus salivarius*

To study the effects of *S. salivarius* colonization in the oral cavity, we cocultured them with the malodor causing bacteria using a millicell insert and observed the cultures under a phase‐contrast microscope. Compared to a single culture of *P. gingivalis*, the coculture with *S. salivarius* saw a rapid decrease in the number of *P. gingivalis* (Figure 4).

**Figure 5:**
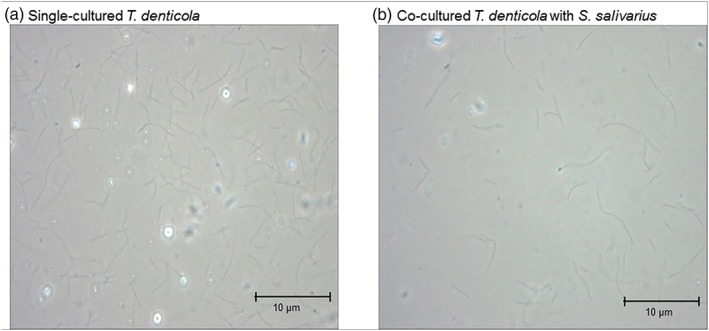
Phase‐contrast microscopic image of single‐cultured or cocultured *Treponema denticola* with *Streptococcus salivarius*

Compared to a single culture of *T. denticola*, the coculture with *S. salivarius* saw a rapid decrease in the number of *T. denticola* (Figure 5).

**Figure 6:**
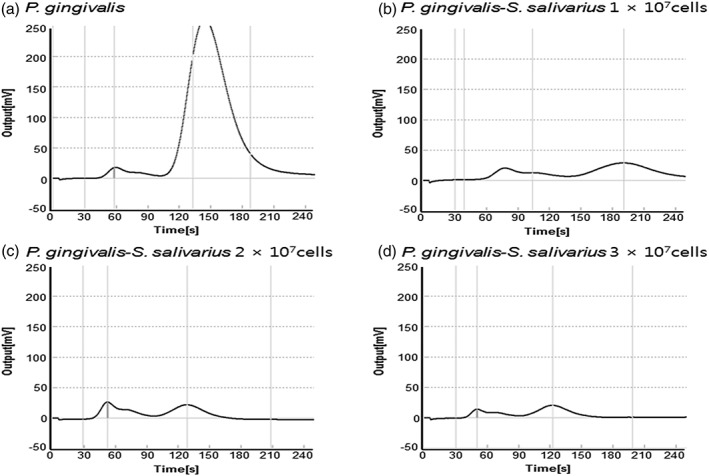
Oral Chroma graphs of single cultures of *Porphyromonas gingivalis* or cocultures with *Streptococcus salivarius*

**Figure 7:**
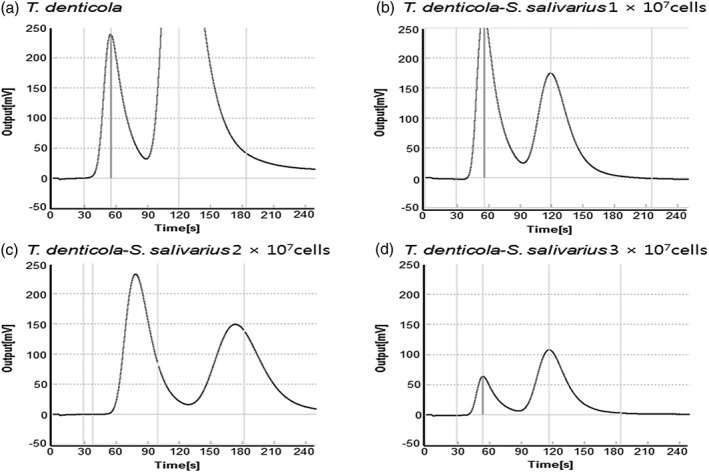
Oral Chroma graphs of single cultures of *Treponema denticola* or cocultures with *Streptococcus salivarius*

When oral malodor causing bacteria such as *P. gingivalis* and *T. denticola* were single cultured, the concentrations of VSCs were higher than when they were cocultured with *S. salivarius* (*p* < .05). As the concentrations of *S. salivarius* K12 and M18 increase, the occurrence of VSCs from oral malodor causing bacteria such as *P. gingivalis* and *T. denticola* decreases (*p* < .05) (Figures 6 and 7).

## 4 DISCUSSION

Oral malodor is an unpleasant smell that occurs in the oral cavity and in nearby organs. The prevalence has increased in modern society, which attaches an importance to social relationships. Bosy (1997) reported that the prevalence of oral malodor among adult's ranges from 25 to 50%, and 25% of afflicted individuals indicated that severe malodor affected their social lives. The American Dental Association reported that 50% of American adults complain of having oral malodor and about 25% have chronic oral malodor (ADA Council on Scientific Affairs, 2003). Kim and Cho (2011) reported that 45% of Korean people and 54% of teenager's desire treatment for oral malodor.

The causes of oral malodor arising from within the oral cavity include biofilms such as dental plaque and tongue coating as well as periodontal diseases. Especially, oral malodor comes from VSCs produced by anaerobic Gram negative bacteria inside biofilms (Amou, Hinode, Yoshioka, & Grenier, 2013; Kato, Yoshida, Awano, Ansai, & Takehara, 2005). Mitsuo et al. (2012) reported finding *P. gingivalis*, *T. forsythia*. and *P. intermedia* in the tongue coating of an individual with a healthy periodontium and they showed a correlation with the concentration of VSCs. On the other hand, *T. denticola* exists in dental plaque and their colonization is related to the concentration of VSCs (Paik et al., 2011). Tanaka et al. (2004) reported that *P. gingivalis*, *T. forsythia*, *T. denticola*, *P. intermedia*, and *P. nigrescens* living in the abdominal area of the tongue have a large effect on the synthesis of VSCs, and that patients with oral malodor showed a higher level of *T. forsythia* than healthy people. They also showed that the ratio of *P. intermedia* and *P. nigrescens* is related to hydrogen sulfide, and that *P. gingivalis* and *P. nigrescens* are associated with methyl mercaptan (Tanaka et al., 2004).

The prevalence of oral malodor shows an increasing trend but treatment methods have been limited to the improvement of oral hygiene involving the physical removal of biofilms by brushing the teeth and the tongue or chemical methods involving mouth wash (Broek, Feenstra, & Baat, 2007). Recently, however, many studies have reported the oral malodor decreasing effects of probiotics. Burton et al. (2005) described the possibility of decreasing oral malodor by using *S. salivarius*. As a result, a new treatment for controlling oral malodor causing bacteria through the use of the probiotic bacteria was proposed. *S. salivarius* can be used as a targeting system to remove harmful bacteria while adhering to the dorsal side of the tongue. They can also secrete a large amount of bacteriocins through saliva. In addition, *S. salivarius* can inhibit the synthesis of VSCs by blocking the colonization of bacteria responsible for synthesizing VSCs. However, the bacteria for which colonization was blocked to decrease oral malodor were unknown (Burton et al., 2005). Park, Auh, Chun, and Hong (2009) reported that *S. salivarius* showed inhibitory effects on *P. intermedia*, which cause periodontal diseases and oral malodor. Lee and Baek (2014) showed that both *L. casei* and *L. rhamnosus* showed inhibitory effects against VSCs synthesized by bacteria that cause periodontal diseases, such as *P. gingivalis* and *F. nucleatum*.

In this study, when *S. salivarius* was cocultured with oral malodor causing bacteria that produce VSCs such as *P. gingivalis*, *T. denticola*, *T. forsythia*, and *F. nucleatum*, there was a statistically significant decrease in oral malodor. In addition, as the concentration of probiotics increased, the decrease in oral malodor became more prominent.

*S. salivarius* K12 and M18 inhibit the synthesis of VSCs from oral malodor causing bacteria such as *P. gingivalis* and *T. denticola*.

Figures 6 and 7 show that when oral malodor causing bacteria such as *P. gingivalis*, *T. denticola*, *T. forsythia*, and *F. nucleatum* were single cultured, the concentrations of VSCs were higher than when they were cocultured with *S. salivarius* (*p* < .05). As the concentrations of *S. salivarius* K12 and M18 increase, the occurrence of VSCs from oral malodor causing bacteria such as *P. gingivalis* and *T. denticola* decreases (*p* < .05).

To investigate the mechanism behind the decrease of oral malodor, we used media containing *S. salivarius* to test for the minimum inhibitory concentration (50%). The results showed antimicrobial activity above a certain level of concentration. From these results, it can be inferred that the oral malodor decreasing effects of *S. salivarius* come from inhibiting the growth of oral malodor causing bacteria.

In the coculture of *P. gingivalis*, *T. forsythia*, and *F. nucleatum* with *S. salivarius*, there was a statistically significant decrease. However, the single cultures of oral malodor causing bacteria showed statistically significant decreases within a smaller range.

From these results, it can be inferred that *S. salivarius* shows a superior effect of reducing oral malodor through antimicrobial activity against oral malodor causing bacteria and the neutralization of sulfur compounds through the colonization of probiotic bacteria.

When *S. salivarius* K12 and M18 live in the oral cavity at concentrations above a certain level, they show antimicrobial activity against oral malodor causing bacteria, reducing the synthesis of VSCs. Using *S. salivarius* K12 and M18 as a treatment to clinically decrease oral malodor is a suitable application.

## 5 CLINICAL RELEVANCE

### 5.1 Scientific rationale for the study

This study provides information that *S. salivarius* inhibits halothane by inhibiting compound synthesis of halitosis‐inducing bacteria.

### 5.2 Principal findings

*S. salivarius* was incubated with the bad breath inducing strain, and the bad breath suppression was prominent with increasing concentration of probiotics.

### 5.3 Practical implication

Clinically, *S. salivarius* K12 and M18 can be used as therapeutic agents for halitosis suppression.
